# Afferent Neuronal Control of Type-I Gonadotropin Releasing Hormone Neurons in the Human

**DOI:** 10.3389/fendo.2013.00130

**Published:** 2013-09-20

**Authors:** Erik Hrabovszky, Zsolt Liposits

**Affiliations:** ^1^Laboratory of Endocrine Neurobiology, Institute of Experimental Medicine, Hungarian Academy of Sciences, Budapest, Hungary; ^2^Department of Neuroscience, Faculty of Information Technology, Pázmány Péter Catholic University, Budapest, Hungary

**Keywords:** GnRH neurons, LHRH, kisspeptin, afferent, humans, hypothalamus, infundibular nucleus, reproduction

## Abstract

Understanding the regulation of the human menstrual cycle represents an important ultimate challenge of reproductive neuroendocrine research. However, direct translation of information from laboratory animal experiments to the human is often complicated by strikingly different and unique reproductive strategies and central regulatory mechanisms that can be present in even closely related animal species. In all mammals studied so far, type-I gonadotropin releasing hormone (GnRH) synthesizing neurons form the final common output way from the hypothalamus in the neuroendocrine control of the adenohypophysis. Under various physiological and pathological conditions, hormonal and metabolic signals either regulate GnRH neurons directly or act on upstream neuronal circuitries to influence the pattern of pulsatile GnRH secretion into the hypophysial portal circulation. Neuronal afferents to GnRH cells convey important metabolic-, stress-, sex steroid-, lactational-, and circadian signals to the reproductive axis, among other effects. This article gives an overview of the available neuroanatomical literature that described the afferent regulation of human GnRH neurons by peptidergic, monoaminergic, and amino acidergic neuronal systems. Recent studies of human genetics provided evidence that central peptidergic signaling by kisspeptins and neurokinin B (NKB) play particularly important roles in puberty onset and later, in the sex steroid-dependent feedback regulation of GnRH neurons. This review article places special emphasis on the topographic distribution, sexual dimorphism, aging-dependent neuroanatomical changes, and plastic connectivity to GnRH neurons of the critically important human hypothalamic kisspeptin and NKB systems.

## Introduction

In all mammals, type-I gonadotropin releasing hormone (GnRH) synthesizing neurons form the final common output way from the hypothalamus in the neuroendocrine control of reproduction. The pulsatile release of GnRH into the hypophysial portal circulation (1, 2) drives the synthesis and secretion of follicle stimulating hormone (FSH) and luteinizing hormone (LH) from anterior pituitary gonadotrophs (3). Circulating FSH and LH, in turn, stimulate gametogenesis and the synthesis and secretion of the gonadal steroid hormones, testosterone from the testes and 17β-estradiol (E2) and progesterone from the ovaries. Under various physiological and pathological conditions, hormonal and metabolic signals regulate GnRH neurons directly and also act on upstream neuronal circuitries to influence the pattern of episodic GnRH secretion. Neuronal afferents to GnRH cells convey important metabolic-, stress-, sex steroid-, lactational-, circadian, and other signals to the reproductive axis. Our knowledge about these inputs is based primarily on the results of physiological and anatomical studies of laboratory species, in particular, rodents. Although it is tempting to assume that the most critical central mechanisms of reproduction are evolutionarily conserved, the direct extrapolation from the results of animal experiments would overlook many obvious species differences already revealed in the hypothalamic regulation of reproduction.

In this article we give an overview of the relatively few neuroanatomical reports that described the afferent regulation of human GnRH neurons by peptidergic, monoaminergic, and amino acidergic neuronal systems. The physiological interpretation of these human neuroanatomical results will be given in the light of experimental data from laboratory species. A special emphasis of the review will be placed on recent data about central kisspeptin (KP) and neurokinin B (NKB) signaling to GnRH neurons. Human genetics provide evidence that central peptidergic signaling by these two neuropeptides play particularly important roles in puberty onset and later, in the sex steroid-dependent feedback regulation of GnRH neurons. We will address the topographic distribution, sexual dimorphism, aging-dependent neuroanatomical changes, and plastic connectivity to GnRH neurons of the human hypothalamic KP and NKB systems. Finally, some critically important remaining questions will be addressed.

## Anatomy of Human GnRH Neurons

### Origin and topographic distribution: Immunohistochemical and *in situ* hybridization results

In mammals, GnRH neurons originate in the olfactory placode and migrate prenatally into the forebrain along the olfactory-vomeronasal nerves (4, 5). In humans, this migratory process is impaired in patients with Kallmann syndrome (6), characterized by hypogonodotropic hypogonadism and anosmia. Species differ significantly regarding the final distribution of GnRH neurons. While the location of GnRH neurons in the most extensively studied laboratory rats and mice is confined to the septal-preoptic region (7, 8), other species including the sheep (9), the guinea pig (10), the ferret (11), the bat (11), or the monkey (11) also have a distinct GnRH cell populations more caudally in the mediobasal hypothalamus/arcuate nucleus (ARC). In addition to a relatively loose distribution of the GnRH cell bodies which quite often lie outside the anatomical borders of classical hypothalamic nuclei, the anatomical definition of the human GnRH neuronal system is complicated somewhat further by the use of different nomenclature in various anatomical reports using *in situ* hybridization (12) and immunohistochemistry (11, 13–25). Based on combined results of these studies, the majority of GnRH neurons in the human are located within a 2 mm wide periventricular zone and show decreasing numbers in the mediolateral direction. Labeled somata are scattered within the septal region, the diagonal band of Broca, the preoptic region, the periventricular hypothalamic nucleus, the mediobasal hypothalamus (infundibular nucleus, Inf and infundibular stalk, InfS), the nervus terminalis, and olfactory regions. *In situ* hybridization studies revealed an additional neuron population expressing intermediate levels of the GnRH transcript (12). These neurons (termed type-III GnRH neurons) exhibit very large perikarya (>500 μm^2^ profile area). Many of them occur at sites not closely related to reproduction (basal nucleus of Meynert, the sublenticular substantia innominata, or the putamen). Because these regions are devoid of GnRH-immunoreactive (IR) somata, type-III GnRH neurons are unlikely to fully process the prohormone to the mature GnRH decapeptide and their role, if any, in reproduction is questionable.

### Morphology of GnRH neurons

As in a variety of other mammalian species (11), the majority of human GnRH neurons are fusiform (12), with thin cell bodies and two processes emanating from the opposite poles of the neurons. A smaller subset of GnRH neurons is multipolar, with triangular or rounded cell body (15). Of note, while similar multipolar GnRH neurons were not observed in the first neuroanatomical studies of rodents, recent morphological characterization of biocytin-filled GnRH-green fluorescent protein (GFP) neurons revealed that 25% of mouse GnRH neurons also have three or more dendrites, in addition to 15% that are unipolar and 65% that are bipolar (26).

### Efferent projections

The postinfundibular eminence of the human hypothalamus plays a pivotal role in the secretion of releasing and release-inhibiting hormones (27). This anatomical site contains a superficial and a deep capillary plexus, both of which are drained into the hypophysial portal system and partly, also to the general circulation (27). Capillaries of both capillary networks are surrounded by GnRH-IR axons (22, 28), suggesting that the superficial and the deep vascular plexuses represent release sites for the hypophysiotropic GnRH axon terminals. It is important to note that the anatomical route of GnRH neurosecretion was reported to show considerable species variations. While in rats, most GnRH axons terminate in the external zone of the median eminence (7), in ferrets and bats a large subset enter the InfS and descend for a considerable distance (11). Similarly, the hypophysiotropic GnRH projections in the human and the monkey hypothalamus enter the InfS and travel all the way down to the neurohypophysis (11, 22). It is reasonable to assume that some GnRH secreted from these terminals reaches the adenohypophysis via the short portal veins.

In addition to hypophysiotropic axon projections, GnRH fibers innervate the organum vasculosum laminae terminalis (OVLT; although in much lower numbers than in the rat) (11), and the subfornical area (13). In the posterior hypothalamus, GnRH fibers surround the mamillary bodies (15). These fibers may already belong to a descending pathway described in various species which traverses the medial forebrain bundle to the ventral tegmental area and midbrain central regions (11). Other extrahypothalamic projection sites present at least in monkeys, include the habenula (29, 30) and the amygdala (31), whereas GnRH projections to the hippocampus and the cingulate cortex have been better characterized in subprimates (11). Ultrastructural evidence exists from both rodents (32, 33) and non-human primates (34) that the non-hypophysiotropic axons of GnRH neurons establish synaptic connections with other neurons.

### Network connectivity of GnRH neurons

The generation of rhythmic secretory bursts is assumed to require the coordinated secretory activity of GnRH neurons, although the underlying mechanisms are still poorly understood. The episodic secretion of GnRH from the GT1-7 GnRH cell line indicates that pulsatility may be an intrinsic property of GnRH neurons in which voltage-gated calcium channels and the connectivity of GT1-7 cell via gap junctions play important roles (35). Classical synapses interconnecting GnRH neurons have been detected in rats (32, 33) and also observed in non-human primates (34). Axo-somatic and axo-dendritic contacts between human GnRH-IR elements (15) suggest that similar synapses also exist in the human hypothalamus, although ultrastructural evidence for this is missing. An additional type of communication has been observed in rats and monkeys whose GnRH neurons form direct dendritic and somatic appositions with one another. At the ultrastructural level, regions of cytoplasmic confluence can be found at some of these contact sites (36). Occasional end-to-end dendritic continuity between neighboring pairs of GnRH neurons has also been observed with light microscopy in the human, indicating syntitium-like associations within a small subset of GnRH neurons (15). This type of connection may have its origins in the nose-to-tail orientation of migrating GnRH cells during embryogenesis (4). Recently reviewed (37) evidence from mice indicates that the dendrites of GnRH neurons form the morphological substrates for a critically important neural communication within the GnRH network. The majority of GnRH dendrites form bundles and intertwine with one another (38). Directly apposed dendrites often receive shared synaptic inputs (38) which may convey excitatory neuronal signals simultaneously to several dendrites. The dendritic tree of mouse GnRH neurons in the vicinity of the OVLT might be more complex than assumed previously. These processes appear to branch extensively in the OVLT which lies outside the blood-brain-barrier and hence, may be influenced by molecules circulating in the bloodstream (39). The synchronization of the GnRH network at the level of dendrites is a newly emerging possibility which gains importance from the observation that dendrites represent the primary sites of action potential generation in the mouse GnRH neuron (40). A final possibility for GnRH neurons to interact with one another is in the median eminence where GnRH terminals may communicate via additional autocrine signaling mechanisms.

### The GnRH pulse generator

The human GnRH pulse generator has been localized to the mediobasal hypothalamus. Explants of this region from fetal and adult human brains release GnRH in a pulsatile manner (41). Recently accumulated evidence gave rise to the hypothesis that the mediobasal hypothalamus in several species contains putative pacemaker neurons that regulate the GnRH neurosecretory pulses. At least in some species, these cells (“KNDy neurons”) co-synthesize KP, NKB, and dynorphins (42–44), whereas a subset of them in the human co-synthesize KP, NKB, and Substance P (SP) (45).

## Neuronal Interactions of GnRH Neurons

### Axo-somatic and axo-dendritic inputs to GnRH cells

Results of ultrastructural studies in rats (36, 46) and monkeys (36, 47) established that GnRH neurons are innervated relatively sparsely, compared with other unidentified neurons located in the same anatomical regions. Quantitative analysis of serial ultrathin sections identified 2–12 synapses per GnRH neuron in male rats and 2–8 synapses in ovariectomized or estrogen-treated female monkeys (36). In post mortem human tissue samples, the relatively poor preservation of the ultrastructure only enables light- and confocal microscopic analyses of axo-somatic and axo-dendritic afferent contacts. Given that the resolution of these methods does not allow the identification of synaptic specializations, many of the observed contacts (juxtapositions) might not be actual synapses. Previous efforts to quantitatively assess the abundance of afferents could thus overestimate some of the specific inputs. Even with this in mind, monoaminergic, and several types of peptidergic neuronal contacts only occur sparsely on human GnRH neurons. A previous review (25) of neuropeptide Y (NPY), SP, beta-endorphin, leu-enkephalin, corticotrophin-releasing hormone (CRH), and galanin containing, and tyrosine hydroxylase (TH)-IR, catecholaminergic inputs reveals general trends in the innervation pattern of single subclasses of GnRH neurons. Accordingly, while GnRH neurons in the septal area do not receive inputs from the above systems and GnRH neurons in the posterior hypothalamus are innervated occasionally only, the heaviest innervation of GnRH neurons can be observed in the infundibulum, followed by the preoptic area. In other studies, only a subset (about 50%) of GnRH-IR neurons were found to receive histaminergic appositions (48) and similarly, KP-IR appositions were only observed on 54% of GnRH neurons in the Inf of young men (49). Even GnRH neurons of the infundibular region with the heaviest SP-IR input were typically innervated lightly; 45% did not receive SP-IR appositions, 37% received only 1–3 contacts and only 18% received more than three contacts per perikaryon (25, 50). Out of the afferent systems of human GnRH neurons studied so far, the most abundant inputs seem to be given off by γ-aminobutyric acid (GABA)ergic and glutamatergic neurons. The mean incidence of light microscopic contacts established by GABAergic axons onto GnRH-IR somata was 45, whereas the VGLUT1 and VGLUT2 subclasses of glutamatergic axons established about 15 and 23 contacts, respectively, per GnRH-IR soma. Amino acidergic contacts were also encountered frequently on the dendrites of GnRH neurons where the combined incidence of glutamatergic contacts (over six contacts/10 μm GnRH dendrite) somewhat exceeded the incidence of GABAergic appositions (over five contacts/10 μm GnRH dendrite) (51). It is important to emphasize that synaptic communication may not occur at a considerable subset of these light microscopic contacts.

The published data, overall, suggest that the innervation of human GnRH neurons is similarly sparse in the human as reported in rodents and monkeys (except for the relatively abundant amino acidergic inputs). On the other hand, immunohistochemical studies could also underestimate the real abundance of synaptic inputs (especially, on somatic and dendritic spines), considering that GnRH immunoreactivity does not necessarily visualize all postsynaptic sites that belong to GnRH neurons. Recent studies of biocytin-filled mouse GnRH neurons provide evidence for dendritic and somatic spines (26), which may provide additional postsynaptic membrane surfaces for excitatory inputs, as elsewhere in the central nervous system (52). Assuming the existence of similar GnRH-immunonegative somatic and dendritic spines in the human, the density of inputs, especially by excitatory afferents that preferentially target the dendritic spines, might be more abundant than indicated by the light microscopic immunohistochemical images.

### Axo-axonal communication

Immediate axonal contacts on GnRH-IR hypophysiotropic fibers have been observed with confocal or electron microscopy in the median eminence of several species. Given that such axo-axonal contacts typically lack synaptic specializations at the ultrastructural level (53, 54), their significance may be difficult to appreciate, unless also supported by receptor localization and/or functional data. For example, GnRH terminals in the median eminence of the rat are apposed to glutamatergic axons (54, 55), contain the KA2 and NR1 ionotropic glutamate receptor subunits (54) and glutamatergic drugs induce Ca^2+^-dependent GnRH release from median eminence fragments in rats (56), indicating together the existence of paracrine communication between glutamatergic and GnRH axon terminals. Many peptidergic systems may also be involved in similar axonal regulation of GnRH secretion. Direct appositions exist between NKB-IR and GnRH-IR axons (53, 57) of the rat which latter express immunoreactivity for the NKB receptor NK3 (57). Furthermore, KP-IR fibers are also apposed to GnRH-IR axons in the goat (58) and the monkey (59) median eminence and such appositions appeared to be frequent compared with axo-somatic and axo-dendritic inputs.

Although interactions at the level of the GnRH axon likely have high functional significance, there are only relatively few reports on similar axo-axonal appositions in the human postinfundibular eminence. We found that KP-IR axons establish such axo-axonal contacts on GnRH-IR fibers in humans (60) and many of these KP fibers also contain NKB and SP (45), increasing further the complexity of possible autocrine/paracrine interactions in the human postinfundibular eminence. While the presence of KP receptor (KISS1R) on GnRH terminals has not been demonstrated formally, there is abundant indirect evidence both from animal and human studies to suggest that the GnRH terminal is an important physiological site of KP action via KISS1R in the regulation of pulsatile GnRH secretion. (i) First, KP can stimulate GnRH release from median eminence fragments of wild type, but not *Kiss1R* mutant, mice (61). This *in vitro* action of KP is independent of action potential generation as it persists in the presence of tetrodotoxin (61). (ii) Second, *in vivo* release of KP into the monkey median eminence is pulsatile and KP peaks coincide with the LH secretory pulses (62). (iii) Third, peripheral injection of KP, which does not cross the blood-brain-barrier, results in a rapid increase of LH release (63–67), suggesting that KP acts on GnRH projections in circumventricular organs. Characterization of axonal neuropeptide receptors will be required in order to clarify the putative autocrine and paracrine interactions that regulate pulsatile GnRH secretion. Unfortunately, the immunohistochemical detection of receptor proteins in *post mortem* tissue samples is most often compromised by the suboptimal tissue processing conditions.

### Efferent targets

Ultrastructural evidence exists from both rodents (32, 33) and monkeys (34) that GnRH-IR axons establish synaptic contacts with other GnRH neurons; these synapses exhibit symmetric specialization. GnRH/GnRH contacts were also observed in the human with the light microscopic analysis of immunolabeled specimens (15).

Relatively little information is known about further postsynaptic target neurons of GnRH-IR axons. Light microscopic observations indicate that the communication between galaninergic and GnRH neurons in the human hypothalamus is bidirectional. The incidence of GnRH-IR inputs to galanin-IR neurons was reported to be highest in the infundibular region (68).

## Neuropeptides and Co-Transmitters in GnRH Neurons

Classical neurotransmitters and neuropeptides co-contained in GnRH neurons may considerably modulate GnRH secretion from hypophysiotropic GnRH axon terminals and/or influence the hypothalamic and extrahypothalamic synaptic communication of GnRH-IR axon projections.

While prenatally, rodent GnRH neurons exhibit immunoreactivity to γ-aminobutyric acid (69), in adult rats GnRH neurons express the mRNA and protein of the glutamatergic marker type-2 vesicular glutamate transporter (VGLUT2) (70). A previous study in our laboratory also addressed this putative glutamatergic phenotype of human GnRH neurons but found no evidence for VGLUT2 immunoreactivity in GnRH-IR axon terminals in the InfS (51). This negative colocalization result may have both biological and technical explanations and the amino acid phenotype of human GnRH neurons remains to be established.

In laboratory animals, GnRH neurons were also found to contain neuropeptide co-transmitters/neuromodulators. A high degree of colocalization has been observed between galanin-IR and GnRH-IR neuronal elements of the rat (71, 72), mouse (73), and sheep (74). The coexpression of galanin in rat GnRH neurons is highly sexually dimorphic and estrogen-dependent (75). Intrinsic galanin may partly act on GnRH neurons via Gal-R1 autoreceptors expressed in GnRH neurons (76). In addition to containing galanin, GnRH neurons in the rat also express cholecystokinin and neurotensin immunoreactivities in sexually dimorphic manners (77). In contrast, the galanin colocalization phenomenon has not been observed in the human hypothalamus (68). Moreover, no successful neuropeptide colocalization has been reported for human GnRH neurons to date.

## Estrogen Signaling to GnRH neurons

Changing levels of the ovarian sex steroid hormone E2 regulate the activity of the neuroendocrine reproductive axis through feedback actions to GnRH neurons (78). In 1983, Shivers et al. (79) reported that GnRH neurons of the rat do not accumulate tritiated E2 *in vivo*. Moreover, subsequent immunohistochemical studies also failed to detect the classical “α” form of estrogen receptor (ERα) in GnRH neurons (80, 81). These observations formed the basis of a prevailing view that most actions of estrogens are communicated to the GnRH neuronal system by estrogen-sensitive neuronal and/or glial elements.

### Direct estrogen sensitivity

Soon after the discovery of a second ER isoform (ERβ) in 1996 (82), the absence of direct estrogen feedback to GnRH neurons was challenged by the observations of ERβ mRNA expression (83, 84), ERβ immunoreactivity (85, 86) and ^125^I-estrogen-binding sites (83) in rodent GnRH neurons. The finding of ERβ, but not ERα, transcripts in rodent GnRH neurons using RT-PCR (87) and *in situ* hybridization (83) techniques suggested that ERβ is the dominant ER isoform involved in direct estrogen feedback to GnRH neurons. This conclusion does not seem to be valid only to rodents, in view that immunohistochemical studies also localized ERβ to a large subset of ovine GnRH neurons (88) which, similarly to the GnRH neurons in other species, lack ERα immunoreactivity (89).

Dual-label immunohistochemical studies from our laboratory also identified ERβ immunoreactivity in 11–28% of GnRH neurons in adult human males (90). In the light of the earlier *in situ* hybridization evidence for the absence of detectable ERα mRNA levels in these cells (90), direct estrogen actions onto human GnRH neurons might be mediated exclusively by the ERβ receptor form.

Functional evidence mostly exists from rodents in support of direct ERβ mediated estrogen effects onto GnRH neurons, as reviewed recently (91). For example, upon ligand binding, ERβ can cause rapid phosphorylation of cAMP-response element-binding protein (91, 92) and stimulate intracellular calcium oscillations (93) via exerting non-genomic effects on mouse GnRH neurons. In addition, ERβ mediates a ligand-independent transcriptional activation and a ligand-dependent transcriptional repression of the mouse GnRH promoter in *in vitro* transfection experiments (94). Furthermore, ERβ-selective agonists can induce galanin mRNA expression in GnRH cells of ovariectomized rats *in vivo* (95), possibly via an ERβ-mediated direct action. The contribution of ERβ-mediated direct estrogen effects to the regulation of human GnRH neurons (90) requires clarification.

It has to be emphasized that although the selective presence of the ERβ isoform in GnRH cells appears to be conserved in different mammalian species, the indirect effects of E2 that are mediated to GnRH cells by ERα containing interneurons clearly dominate in the regulation of reproduction over the ERβ-mediated direct effects. Accordingly, both negative and positive estrogen feedback mechanisms are disrupted in the ERα-knockout mice (96) and human case reports also exist to show that the disruption of ERα signaling causes profound estrogen resistance in women, with delayed puberty and the absence of menstrual cyclicity (97).

### Estrogen-sensitive interneurons of the infundibular region

The hypothalamic ARC (Inf) has long been considered as an important site of sex steroid negative feedback to the reproductive axis. The absence of this negative estrogen feedback in postmenopausal women causes profound anatomical changes in the human Inf, with a robust hypertrophy of ERα (24), SP (98), NKB (98), KP (99), and prodynorphin (100) expressing cells. Morphological alterations are accompanied by increased NKB (98)-, KP (99)-, and SP (98)- and decreased prodynorphin (100) mRNA synthesis. The high levels of KP and NKB immunoreactivities detectable in the Inf of postmenopausal women (101) support the notion that the inhibitory control of KP and NKB synthesis in these cells is diminished in the absence of E2.

Recent experimental evidence from animal studies confirm that the role of KP/NKB neurons is critical in gonadal steroid feedback to GnRH neurons in both sexes and in various species. Of note, the ablation of these cells in rats with locally applied microinjection of the NK3-saporin neurotoxin prevented the rise in serum LH and attenuated the rise in serum FSH following ovariectomy (102). The estrogenic suppression of gonadotropin secretion has not been entirely blocked in the lesioned rats, indicating also some redundancy of the neuronal pathways mediating the negative feedback effects of estrogens (102). It is worth to note that the ablation of the same KP/NKB cell group in the ARC also interfered with the estrogenic suppression of body weight (102). Furthermore, these cells appear to also convey important estrogen signals to the thermoregulatory centers. It is hypothesized that the dysregulation of this signaling during menopausal transition is also involved in the pathogenesis of hot flushes (103).

### Possible role for the rostral periventricular region

Recent immunohistochemical work from our laboratory identified a relatively compact KP cell population in the rostral periventricular region of premenopausal women but not in men (60). The distribution of these neurons overlapped with the ventral periventricular nucleus, the anterior parvicellular paraventricular nucleus and the parvicellular and magnocellular subdivisions of the paraventricular nucleus, according to the anatomical atlas of Mai et al. (104). A more thorough characterization of this cell population will require the further investigation of tissues samples from male and female individuals of different ages.

Spontaneous menstrual cyclicity and LH/FSH responses to estrogen are well-preserved after the mediobasal hypothalamic deafferentation of non-human primates (105) and E2 can also elicit gonadotropin surges after acute complete removal of the neural tissue dorsal and anterior to the optic chiasm (106). These data support the prevailing view that positive sex steroid feedback in primates takes place exclusively in the infundibular region (107). The presence of a sexually dimorphic nucleus and KP cell group in the human rostral periventricular region raises a challenge to this view.

## Afferent Inputs to GnRH Cells

### Neuropeptides

#### Neuropeptide Y

The 36-amino acid peptide NPY acts at multiple levels of the reproductive axis to influence gonadotropin secretion. At the hypophysial level, it potentiates LH secretion in response to a GnRH challenge by modulating GnRH binding to anterior pituitary GnRH receptors (108). At the level of the median eminence, it stimulates GnRH secretion from GnRH axon terminals (109, 110). Finally, an important site of central NPY actions might be on the somato-dendritic compartment of GnRH neurons. The majority of GnRH neurons in the rodent preoptic area receive abundant innervation from NPY-containing axon terminals (111, 112) and express the Y5 receptor form (113). At least two separate sources exist in rodents for the NPY-IR inputs to GnRH neurons. Retrograde labeling of NPY neurons has been observed in both the ARC and the ventrolateral medulla following retrograde tracer injection around GnRH neurons (114). In a previous study from our laboratory we used two site-specific neurochemical markers to determine the relative contribution of these two cell populations to the innervation of GnRH neurons (115). About half of the NPY-IR contacts on GnRH cells also contained agouti-related peptide, indicating their origin in the ARC where the two peptides are co-synthesized in the same neurons (116). A further one-fourth of the contacts contained the noradrenergic/adrenergic marker enzyme dopamine-β-hydroxylase (115), indicating the origin of these inputs in brainstem catecholamine cell groups (117). The source of the remaining one-fourth of the NPY fibers making contacts with GnRH neurons, requires clarification.

Neuropeptide Y plays an important role in the central regulation of the reproductive axis, in particular, in the metabolic regulation of fertility (118). While a chronic increase in NPY tone inhibits gonadotropin release (119), delays sexual maturation (120), and suppresses estrous cyclicity (121), the direction of acute NPY effects depends markedly on the sexual steroid status. For example, while in castrated animals, central administration of NPY decreases gonadotropin secretion (122, 123), in intact rodents or gonadectomized and steroid-primed rodents, NPY increases serum levels of gonadotropins (119, 124). Among the multiple and potentially redundant peptidergic pathways that mediate metabolic effects on the reproductive axis (118), the functional significance of the direct NPY input to GnRH neurons requires clarification.

In the human, NPY-IR varicosities establish similar axo-somatic and axo-dendritic contacts with GnRH-IR cell bodies and dendrites as in rodents. Individual GnRH neurons are often contacted by multiple axonal swellings (15). Juxtapositions are most numerous in the Inf, followed by the medial preoptic region and then, the anterior periventricular nucleus (15). The sources of NPY axons innervating human GnRH neurons needs to be determined using similar region-specific phenotype markers as earlier studies on mice (115).

#### Substance P

Substance P is an eleven-amino acid-neuropeptide derived from the preprotachykinin A gene, which exhibits the highest affinity to the G protein-coupled NK1 receptor, whereas neurokinin A, another derivative of the same gene, acts via the NK2 receptor (125). As reviewed recently, SP can influence reproduction at the hypothalamic, pituitary, and gonadal levels of the reproductive axis (126). Previous studies identified both inhibitory and excitatory LH responses to SP. For example, SP can inhibit the GnRH-stimulated secretion of LH from cultured human pituitary cells (127). On the contrary, intravenous, or intracerebroventricular SP injection to ovariectomized estrogen primed rats stimulates, and conversely, intracerebroventricular application of a SP antiserum to ovariectomized rats inhibits LH release (128).

An important site of hypothalamic SP synthesis is the ARC/Inf. The SP system of the rat ARC shows morphological changes across the estrous cycle, with maximal IR cell numbers detected at proestrus/estrus (129, 130). SP-IR axons originating partly from this nucleus form synaptic contacts with GnRH neurons of the preoptic region (131). Preprotachykinin A mRNA expression (98) and SP immunoreactivity (50, 132) have also been revealed in neuronal cell bodies of the human Inf. Similarly to the rat SP neurons, these cells appear to be estrogen-sensitive, as indicated by their hypertrophy and enhanced mRNA expression in postmenopausal women in the absence of estrogens (98).

Substance P neurons of the human infundibular region may influence reproduction via multiple actions, depending on the currently unknown site/s of location of its main receptor, NK1. Important hypothalamic effects may be exerted through SP-IR axo-somatic and axo-dendritic contacts identified on GnRH neurons (50). The NK1 receptors underlying this communication may either be pre- or postsynaptic. SP neurons appear to form a network within the Inf; they may regulate one another via the frequently encountered axo-somatic and axo-dendritic contacts (50) that are reminiscent of a similar networking among the NKB neurons in laboratory animals (133). NKB-signaling through these NKB/NKB contacts use NK3 autoreceptors and this communication has been recently implicated in the mechanism of pulsatile GnRH/LH secretion (42, 43, 134). In humans, SP-IR neurons densely innervate the neurovascular zone of the InfS (132). These projections may be involved in important axo-axonal interactions and may also serve as the major source of SP in the portal circulation to exert adenohypophysial actions (127).

The distribution of SP and NKB neurons in the human Inf is very similar (98). Recent immunohistochemical evidence from our laboratory indicates that considerable subsets of KP-IR (30.6%) and NKB-IR (25.1%) neurons in the Inf of postmenopausal women also contain SP immunoreactivity (45). This interesting colocalization phenomenon has not been reported so far in laboratory animals and thus, might only exist in certain species. The presence of SP in NKB and KP neurons increases the functional complexity of the putative pulse generator network thought to be localized in the ARC/Inf. From the functional view-point, it is possible that SP modulates the effects of KP and NKB in axo-somatic and axo-dendritic afferents to GnRH neurons. Intrinsic SP may also exert autocrine/paracrine actions via autoreceptors to regulate the activity and/or neuropeptide release of NKB/SP and KP/SP neurons. In the InfS, SP may either influence the KP and NKB secretory output via autocrine/paracrine mechanisms or regulate GnRH neurosecretion directly. Finally, possible co-release of SP with KP and NKB into the portal circulation could underlie further interactions on adenohypophysial gonadotrophs.

#### Galanin

Galanin is a 29-amino acid peptide (135). It is widely distributed in the central nervous system and acts as a transmitter, modulator, and growth factor in a number of physiological and disease-related processes (136).

Galanin regulates rodent reproduction partly via acting in the adenohypophysis. First, it is synthesized locally in the anterior pituitary (137). In addition, hypothalamic galanin also reaches gonadotroph cells via the hypophysial portal circulation (138). Galanin is secreted into the portal blood in a pulsatile manner (138) and enhances GnRH receptor binding on gonadotroph cells (139). The main hypothalamic sources of galanin in the portal blood of the rat are the ARC and the paraventricular nucleus (140). The scattered galanin neurons in the medial preoptic area that project to the median eminence correspond to GnRH neurons which express galanin in a sexually dimorphic and estrogen-dependent manner (141).

Galaninergic neurons also regulate reproduction at the level of the hypothalamus in rats (71) and mice (142), partly via providing direct innervation to GnRH neurons.

The anatomical relationship of galanin-IR and GnRH-IR neurons in the human hypothalamus has been studied with dual-label immunohistochemistry. A detailed topographic study localized the majority of galaninergic neurons posteriorly to the lamina terminalis. Densely packed galanin-IR cell bodies were present in high numbers in the infundibulum and were often located around the portal capillary vessels. Numerous IR perikarya were detected at the basal surface of the tuberal region, extending from the Inf to the optic tract. Many galanin-IR perikarya were detected in the periventricular and paraventricular nuclei and in the chiasmatic and tuberal regions (68).

Dual-label immunohistochemical studies of post mortem human hypothalamic sections identified a bidirectional communication between galanin and GnRH neurons (68). Galanin-GnRH and GnRH-galanin juxtapositions were most numerous in the medial preoptic area and in the infundibulum/median eminence (68). These contacts were proposed to represent the major morphological substrate of the galanin-controlled gonadal functions in humans. The issue of which galaninergic cell populations are involved in the innervation of human GnRH neurons requires clarification.

#### Corticotropin-releasing hormone

There is a large body of evidence to suggest that central corticotropin-releasing hormone (CRH) signaling plays a crucial role in the stress-induced suppression of the GnRH pulse generator. For a recent review, see Li et al. (143). Intracerebroventricular administration of CRH inhibits multiunit activity volleys in monkeys (144). It also decreases LH pulse frequency in rats (145). Furthermore, the suppression of LH pulses by insulin-induced hypoglycemic stress can be completely prevented by intracerebroventricular administration of a CRH antagonist (145).

Some of the suppressive effects of central CRH signaling might be exerted directly on GnRH neurons which receive synaptic inputs from CRH-IR structures in the rat hypothalamus (146). The source of this innervation is, however, unclear, in view that CRH-IR axons might arise from multiple brain sites, including the hypothalamic paraventricular nucleus, the bed nucleus of the stria terminalis and the central amygdala, among many other regions.

Juxtaposition of CRH-IR fibers to GnRH neurons has also been observed in the human hypothalamus (147). Similarly to a series of other specific inputs (25), these neuronal contacts were most frequently encountered in the infundibular region (147).

#### Kisspeptin

Kisspeptin signaling via its specific receptor (KISS1R) plays critically important roles in the regulation of puberty and reproduction. Inactivating mutations of the encoding *KISS1* (148) and *KISS1R* (149, 150) genes, respectively, cause hypogonadotropic hypogonadism in humans. Today’s consensus is that KP neurons regulate reproduction mainly via stimulating hypothalamic GnRH secretion. In different mammalian species, GnRH neurons are innervated by KP-IR afferents (59, 60, 151, 152), express *Kiss1R* mRNA (153–155), and respond with Fos expression (63, 154) and depolarization (155–157) to KP.

The topographic distribution, network connectivity, neurochemistry, sexual dimorphism, and aging-dependent morphological plasticity of the human hypothalamic KP neuronal system have been analyzed in a series of recent morphological studies. Both *in situ* hybridization (99) and immunohistochemical (60) studies localized the majority of KP synthesizing neurons to the Inf and the InfS. Immunohistochemical studies of young female subjects also detected a more lightly stained second KP cell group in the rostral periventricular area of the third ventricle (RP3V) (60). A smaller third KP cell population consisting of darkly labeled neurons is scattered periventricularly at all rostro-caudal levels of the medial hypothalamus (60).

The axons of KP neurons establish contacts with the cell bodies, dendrites, and hypophysiotropic fiber projections of GnRH neurons (60). These appositions are thought to represent the primary sites of KP action on the reproductive axis. As mentioned in later sections, the incidence of such appositions shows considerable plasticity, being higher in postmenopausal women compared with age-matched men (101) or in aged, compared with young men (49).

The exact role of the distinct KP cell populations in human reproduction requires clarification. In rodents, the RP3V encloses a higher number of KP neurons in females than in males (152, 158, 159) and there is strong case that these KP neurons are critically involved in positive estrogen feedback to GnRH neurons (160). Both in female rodents (159, 161) and sheep (162, 163) the preoptic KP neurons are activated before the preovulatory GnRH/LH surge. The presence of a sexually dimorphic “RP3V”-like nucleus and KP cell population in humans (60) raises a challenge to the prevailing view that positive sex steroid feedback in primates takes place exclusively in the infundibular region (107).

Independently from the species, KP neurons of the ARC/Inf have been implicated in the negative feedback effects of gonadal steroids to GnRH neurons (164–166) and their neurotoxic ablation in rats, indeed, prevented the rise in serum LH and attenuated the rise in serum FSH following ovariectomy (102). In sheep and possibly in primates, KP neurons of the ARC may also play a role in positive estrogen feedback to GnRH neurons. Both ovariectomy and a surge-inducing E2 treatment could induce Fos expression in KP neurons of the sheep ARC (167). In addition, KP neurons of the hypothalamic ARC/Inf may represent an important constituent of the hypothalamic pulse generator which regulates the episodic secretion of GnRH into the hypophysial portal circulation. Recent models (42, 43, 168, 169) hypothesize that the intranuclear network communication of these KP neurons uses other colocalized neuropeptides and their autoreceptors, whereas KP provides the major output signal toward GnRH neurons.

#### Neurokinin B

Unlike the preoptic KP cell population, KP neurons in the sheep (164, 168), goat (43), mouse (42), and monkey (170) ARC also synthesize the tachykinin peptide NKB. The majority of human KP neurons in the Inf have also been shown to contain NKB immunoreactivity (60), although the inverse colocalization pattern (incidence of KP expression in NKB neurons) in humans shows considerable sex- and age-dependencies (49, 101). Similarly to KP, NKB plays a crucial role in the regulation of puberty and reproduction. Inactivating mutations of the genes encoding for NKB (*TAC3*) or the NKB receptor NK3 (*TACR3*) cause hypogonadotropic hypogonadism in the human (171, 172). KP neurons of the ARC in various mammalian species also contain the NK3 autoreceptor (42, 57, 133, 173). NKB-signaling via the NK3 autoreceptor can significantly modulate the frequency of the GnRH neurosecretory pulses. For example, central administration of NKB increases the frequencies of multiunit activity volleys and LH secretory pulses in ovariectomized goats, likely via acting on this NK3 autoreceptor (43).

The issue of whether or not, NKB influences GnRH neurons directly, is controversial. GnRH-IR axons are apposed to NKB-IR axons in the rat median eminence (53, 57) where GnRH fibers express NK3 immunoreactivity (57). Somewhat conflictingly, mouse GnRH neurons do not show electrophysiological responses to the NK3 agonist Senktide (174).

We note that human NKB neurons in the infundibular region outnumber KP neurons in all human models that we examined so far with immunohistochemistry (28, 49, 60, 101). The largest difference was noticed in young human males where only 33–36% of the NKB neurons expressed KP immunoreactivity (49). This low colocalization percentage then increased to 68% in aged male individuals (49).

Similarly to KP neurons (60), NKB neurons in the human hypothalamus form afferent contacts with the somatic, dendritic, and axonal compartments of GnRH neurons (28, 49, 101). Interestingly, the incidence of NKB-IR contacts onto GnRH neurons is significantly higher than that of KP-IR inputs, and depending on the human model, only 10–30% of the KP-IR and NKB-IR inputs to GnRH neurons were found to be double-labeled for the other peptide (49, 101). The identity of neurotransmitter(s) used in this communication and, the putative involvement of NKB/NK3 signaling, require clarification.

#### Endorphins

Endogenous opioid peptides are important central inhibitors of the reproductive axis. Since the opiate antagonist naloxone had no direct influence on pituitary release of LH and the LH-releasing effect of systemic naloxone treatment could be prevented with GnRH antagonist in rats, the effects of opioid peptides are likely mediated by the GnRH neuronal system (175).

Microinjection of the proopiomelanocortin gene-derived opioid peptide β-endorphin into the mediobasal hypothalamus and the medial preoptic area, the major sites of location of GnRH-IR elements, significantly suppressed LH secretion in rats (176). Conversely, administration of β-endorphin antiserum into these areas elevated LH secretion (177). In contrast, when hemipituitaries of ovariectomized rats were incubated *in vitro* with 10^−7^ M β-endorphin, basal, or GnRH-induced LH release was not suppressed (176). The above data, together lead to the conclusion that β-endorphin acts at a medial hypothalamic and/or preoptic-septal site and not the pituitary, to alter secretion of LH.

At least some of the effects of β-endorphin on GnRH neurons might be exerted through the direct synaptic connectivity of the two systems reported both in juvenile female monkeys (34) and in male rats (33). The authors of the rodent study reported the presence of β-endorphin in 10% of synaptic afferents to GnRH neurons (33).

Light microscopic contacts have also been observed between β-endorphin-IR axons and GnRH-IR neurons in the human where the contacts were most abundant in the infundibular region (178). The β-endorphin-IR input to human GnRH neurons is likely to arise from local sources given that the mediobasal hypothalamus contained a high number of β-endorphin-IR perikarya (178). Interestingly, while a series of immunohistochemical studies showed that GnRH neurons in front of the lamina terminalis do not receive significant input from axons containing catecholamines (179), NPY (15), SP (50), leu-enkephalin (180), and CRH (147), β-endorphin containing axons did innervate this rostral subpopulation of GnRH neurons (178).

#### Enkephalins

The preproenkephalin gene-derived opioid peptides leu-enkephalin and met-enkephalin can modulate pituitary LH release via direct actions at the pituitary level. Although enkephalins alone have no effect on LH release *in vitro*, they can potentiate the LH response to GnRH (181). The effects of enkephalins on the reproductive axis appear to be indirect and mediated by GnRH neurons. Multiple enkephalinergic systems may contribute to this regulation. One of such systems identified in the mouse RP3V co-expresses KP (182). These KP neurons are more numerous in females compared with males (152) and they have been implicated in positive estrogen feedback to GnRH neurons in rodent species (160). The putative existence of an analogous enkephalin/KP cell group in the human rostral hypothalamus awaits clarification.

Direct regulatory actions of central enkephalinergic pathways on human GnRH neurons have been addressed with dual-label immunohistochemical studies (180). These experiments established that Leu-enkephalin-IR cell bodies are concentrated in three different regions: (i) in the periventricular area of the tuberal region, (ii) in the infundibulum in the close proximity of the portal vessels, and (iii) in the medial preoptic area periventricularly (180). Leu-enkephalin-IR fibers established appositions to GnRH-IR cell bodies and dendrites mainly in the infundibulum and the median eminence (180).

#### Dynorphins

Kisspeptin and neurokinin B neurons of the ARC synthesize the opioid peptide dynorphin in the rat, mouse, sheep, and goat (42, 43, 133, 164, 168), serving as the basis for the recently introduced “KNDy neuron” terminology (169). Central injection of dynorphin to ovariectomized goats decreases the frequencies of multiunit activity volleys and LH secretory pulses (43).

The Inf of the human hypothalamus also contains prodynorphin mRNA expressing neurons (100) and the majority of GnRH neurons in the human hypothalamus have close contacts with dynorphin fibers (183). While the existence of triple-neuropeptide phenotype KNDy neurons in the human Inf has been predicted from the animal data, a recent immunohistochemical study from our laboratory only found evidence for scattered KP/dynorphin dual-labeled axons in the Inf of young human male subjects, whereas most KP cell bodies and fibers were devoid of dynorphin immunoreactivity (28). It seems likely that these negative colocalization data reveal an important species difference of the human from laboratories species. Alternatively, partial *post mortem* degradation of dynorphin in KP-IR neuronal elements of the human Inf can not be entirely ruled out. Of note, preliminary data from our laboratory using well-preserved human tissues samples perfusion-fixed within a few hours after death support the view that IR dynorphin A and dynorphin B can only be detected within very small subsets of KP-IR axons in the human infundibular region.

#### RF-amide related peptides/gonadotropin-inhibiting hormone

Gonadotropin-inhibiting hormone (GnIH) plays a crucial role in the inhibitory regulation of the reproductive axis. In birds, GnIH appears to have at least two sites of action on the reproductive axis. First, it directly inhibits gonadotropin release from the pituitary in a dose-dependent manner (184). In addition, it occurs in neuronal afferents that form contacts with GnRH cells (185) which latter, contain receptors for GnIH (186). The putative GnIH homologs, RF-amide related peptides (RFRP-1, RFRP-2, RFRP-3), have been identified in mammals (187). Neurons synthesizing preproRFRP mRNA and RF-amide peptides have been localized mostly to the dorsomedial nucleus of the hypothalamus in hamsters, rats, mice, and sheep (188–193).

While it is generally agreed that GnIH/RFRP neurons in birds, rodents, ewes, and primates can act via regulating GnRH neurons (185, 190, 192–195), the additional adenohypophysial site of action of RFRP peptides in mammals has been controversial. Despite *in vitro* evidence from several species for direct pituitary effects (196–198), GnIH/RFRP-IR terminals have only been observed in the neurosecretory zone of the median eminence in birds (184, 196, 199) and monkeys (200), but not in rodents (190, 192). Furthermore, RFRP synthesizing neurons in rats do not accumulate Fluoro-Gold from the systemic circulation, indicating lack of access to the hypophysial portal circulation (192).

There has only been one morphological report so far addressing the role of RFRPs in human reproduction (200). This study used affinity column purified rabbit anti-white-crowned sparrow GnIH antibodies for the immunohistochemical detection of GnIH-IR structures. This study came to the conclusion that IR cell bodies of the human are located in the dorsomedial nucleus, send axonal projections to GnRH neurons in the preoptic area as well as to the median eminence (200). Furthermore, RT-PCR and subsequent DNA sequencing of the PCR products identified human GnIH receptor (GPR147) mRNA expression in the pituitary and *in situ* hybridization confirmed its expression in LH producing gonadotropes (200). The future use of human RFRP-specific antibodies will be important to reproduce these interesting immunohistochemical findings which suggest that the RFRP system in the human may regulate reproduction both at the hypothalamic and the hypophysial levels.

#### GnRH

In rats (32, 33) and non-human primates (34), GnRH-IR axons establish symmetric synaptic contacts with one another at the ultrastructural level. This communication channel and autocrine/paracrine regulatory mechanisms have been implicated in a putative ultrashort loop feedback mechanism whereby GnRH inhibits the neuronal activity of GnRH neurons. In some studies the incidence of GnRH-IR synaptic inputs to GnRH neurons almost reaches 10% of all synaptic inputs (33).

Human GnRH neurons also establish axo-somatic and axo-dendritic appositions with one another at the light microscopic level (15), which may correspond to synaptic connections.

#### Other peptidergic systems

Many further peptidergic systems exert direct regulatory actions on GnRH neurons in laboratory animals. Some of the specific inputs are derived from lateral hypothalamic peptidergic neurons and their neurotransmitters/neuromodulators include melanin concentrating hormone (194) and orexin (201, 202). Other afferents contain cocaine and amphetamine regulated transcript (203). The existence of analogous neuronal connections in the human hypothalamus will require clarification.

### Monoamines

While the role of serotonin in the afferent control of GnRH neurons has not been addressed in the human, anatomical evidence exists to support the role of catecholamines and histamine in the afferent control of human GnRH neurons.

#### Catecholamines

Different catecholamines exert wide effects on LH release mostly via central actions (204–207), although evidence also exists for the hypophysial site of catecholamine actions (208).

Catecholamines of hypothalamic and extrahypothalamic origins partly modulate gonadal functions via direct interactions with hypothalamic GnRH neurons. Earlier studies on rodents identified appositions between catecholaminergic fibers and GnRH-IR cell bodies in rats (209) and mice (115).

In a previous study (179), the distribution of GnRH-IR and TH-IR structures and their neuronal interactions were also addressed in the human diencephalon. This study identified TH-IR dopaminergic perikarya in the periventricular, paraventricular, and supraoptic hypothalamic nuclei and also in the median eminence. The TH-IR fibers were numerous in septal, infundibular, periventricular, and lateral hypothalamic regions. GnRH neurons in the infundibular and medial preoptic areas received TH-immunopositive axon contacts, whereas GnRH-IR perikarya only received a few juxtapositions in the preoptic area and the caudal parts of the diencephalon. Because TH is also required for the synthesis of epinephrine and norepinephrine in the brain stem, these catecholaminergic inputs could arise from various dopaminergic, adrenergic, and noradrenergic cell groups. The contribution of different catecholamines to the afferent regulation of human GnRH neurons will thus require clarification.

#### Histamine

E2 increases histamine release from hypothalamic tissue blocks *in vitro* (76), histaminergic neurons provide abundant input to the preoptic area (210) which also contains GnRH neurons, intracerebroventricular injection of histamin causes ovulation in rabbits (211) and the immortalized GT1-7 neuronal cell line expresses H1 histamine receptors (212). Based on these data and in search for neuronal systems that might mediate the feedback effects of E2 to GnRH neurons, we investigated the possible role of the histaminergic neuronal system in rats and humans (48).

Results of our studies on rats (48) showed that 66–81% of histaminergic neurons in all subdivisions of the tuberomamillary complex contained nuclear ERα immunoreactivity. Histaminergic axons in the preoptic region established axo-dendritic and axo-somatic contacts with GnRH-IR neurons. Finally, lateral ventricular infusion of the H1 receptor antagonist mepyramin inhibited the estrogen-induced surge of LH, whereas the H2 receptor antagonist ranitidin was without effect.

The putative human relevance of these data was addressed via the immunohistochemical analysis of histaminergic neuronal contacts onto human GnRH neurons. 51 ± 3.0% of GnRH neurons in these studies were found to receive histamin-IR axonal appositions (48). In other studies, both the ERα and the ERβ receptor isoforms have been localized to the human tuberomamillary complex with immunohistochemistry (213). The above data indicate that the positive feedback effect of E2 on the preovulatory LH surge may involve estrogen-receptive histamine-containing neurons within the tuberomamillary regions which communicate with GnRH neurons via H1 receptors.

### Amino acid neurotransmitters

The most abundant inhibitory and excitatory neurotransmitters in the hypothalamus are GABA (214) and glutamate (215), respectively.

#### γ-Aminobutyric acid

Based primarily on evidence from studies of laboratory rodents, GABA is thought to represent the principal neurotransmitter in the synaptic control of GnRH neuronal functions (216). Such studies showed that GnRH neurons receive an abundant GABAergic synaptic input (217), express functional receptors for both the ionotropic GABA_A_ (218–220), and the metabotropic GABA_B_ (221) receptors and all of them exhibit GABA_A_ receptor-mediated postsynaptic currents (219, 220). While the first studies disagreed on whether the currents mediated by the chloride channel GABA_A_ receptors, excite (219), or inhibit (222) adult mouse GnRH neurons, a recently formed consensus opinion (223) is that the dominant effect of GABA_A_ receptor activation is excitation, as also proposed for GABAergic currents in rat GnRH neurons (224). This atypical behavior is due to the absence (or only low expression levels) of the K-Cl co-transporter (KCC2) in GnRH cells (219), an enzyme which normally extrudes chloride from other adult-type neurons. Given that GnRH neuron activity is increased by both GABA and glutamate, these cells need alternative mechanisms for their inhibitory afferent regulation, including retrograde endocannabinoid signaling which inhibits the excitatory GABAergic inputs to GnRH neurons in mice (225).

A recent dual-label immunohistochemical study from our laboratory (51) addressed the relative contributions of GABAergic and glutamatergic afferent neuronal contacts onto the cell bodies and dendrites of human GnRH neurons at the light microscopic level. While an earlier innervation study in the rat used antibodies against glutamic acid decarboxylase to identify GABAergic synaptic afferents to the rat GnRH neurons (217), we detected the GABAergic terminals with a primary antiserum directed against the vesicular inhibitory amino acid transporter (VIAAT) which accumulates GABA (and glycine) into synaptic vesicles.

The results of these studies showed that VIAAT-IR axons establish many axo-somatic and axo-dendritic contacts with human GnRH neurons. The number of such light microscopic appositions was one or two orders of magnitude higher than reported for different peptidergic systems (25), supporting the idea that GABA is also the major neurotransmitter in the afferent regulation of GnRH neurons in the human (51).

#### Glutamate

The invariable occurrence of a metabolic glutamate pool in every cell type has made it difficult to immunohistochemically identify excitatory neurons that use glutamate for synaptic communication. In the absence of selective glutamatergic markers, early immunohistochemical work used antibodies against glutamate *per se*. Such studies provided evidence for IR glutamate in axon terminals that establish asymmetrical synapses with the dendrites, but not the somata, of GnRH-IR neurons in the monkey (226). The use of specific antisera against the vesicular glutamate transporter isoforms (VGLUT1 and VGLUT2), discovered relatively recently, provided new tools to also distinguish between the two major subclasses of glutamatergic afferents to GnRH neurons. Immuno-electron microscopic studies of rats established that GnRH neurons only receive synapses from the VGLUT2 subclass of glutamatergic neurons and these inputs are most prominent on the dendritic compartment of GnRH cells (227).

In contrast with rodent GnRH neurons, we found that human GnRH neurons were contacted by both VGLUT1-IR and VGLUT2-IR fibers, out of which those of the VGLUT2 phenotype were encountered more frequently (51). The combined incidence of these two fibers was somewhat lower on GnRH cell bodies than the incidence of GABAergic contacts, whereas glutamatergic axons (VGLUT1 + VGLUT2) outnumbered GABAergic inputs on GnRH-IR dendrites (51). This glutamatergic dominance is particularly interesting in the light of new information that dendrites are the primary sites of action potential generation in the GnRH neuron, at least in mice (40).

## Regional Heterogeneity in the Afferent Regulation of GnRH Neurons

As summarized in a review article (25), the incidence of various peptidergic (NPY-, SP-, β-endorphin-, leu-enkaphalin-, CRH-, and galanin-IR) and catecholaminergic inputs to human GnRH neurons changes region-specifically. The most heavily innervated GnRH subclasses are located in the infundibulum and the median eminence, followed by GnRH neurons in the preoptic area. GnRH-IR neurons in the posterior hypothalamus were only innervated occasionally. This differential innervation raises the possibility that the human GnRH neuronal system consists of several functional subclasses (25).

## Sexual Dimorphism and Aging-Dependent Changes in the Afferent Control of GnRH Neurons

The majority of neuroanatomical studies addressing the innervation of human GnRH neurons only provide qualitative information based on the analysis of a few histological specimens. Difficulties to access human tissue samples in large number, together with the typically high biological, and technical variations among the samples (partly due to different circumstances of life, death, and tissue preservation) are discouraging for quantitative studies using state-of-the-art statistical methods. The most robust biological differences may still be detectable.

Previous quantitative studies of the human KP and NKB systems in our laboratory included histological samples from three adult human models categorized arbitrarily as “young human males” (below 50 years of age), “aged human males” (above 49 years of age), and aged (postmenopausal) women (above 55 years of age). Perikaryon size (labeled IR profile area), regional density of IR cell bodies and neuronal fibers in the Inf, frequency of IR fiber appositions to GnRH-IR neurons, and the colocalization percentages between KP and NKB were determined and compared with ANOVA.

### Sexual dimorphism of kisspeptin and neurokinin B neurons

In the absence of hypothalamic tissue samples from premenopausal female subjects, sex differences were only assessed between postmenopausal women and aged men (above 49 years) and not between young female and male individuals. Because aged females lack estrogen negative feedback whereas aged males maintain testosterone negative feedback on KP and NKB neurons of the Inf, the sexual dimorphism of these systems might be entirely due to the different sex steroid milieu in the two sexes. Alternatively, females and males may show differences that form under the combined organizational and activational effects of sex steroids. The conceptually important issue of whether or not any sexual dimorphism exists in the human hypothalamus that forms under the organization effects of sex steroids during early development, thus requires clarification.

Larger KP-IR and NKB-IR cell bodies were observed in females than in males (101). The postmenopausal hypertrophy of KP (99) and NKB (98) neurons reported earlier and attributed to the postmenopausal loss of estrogen negative feedback, may partly, or entirely explain these sex differences. KP neurons also showed other robust sex differences in that the number of KP-IR somata, the density of KP-IR fibers and the incidence of afferent contacts they formed on GnRH neurons were much higher in aged women compared with aged men. These differences, together, indicate a robust aging-related expansion of the KP neuronal system and a largely enhanced excitatory KP tone on GnRH neurons of postmenopausal women compared with elderly men.

The regional density of NKB cell bodies was also significantly higher in women compared with men, but there was no significant sex dimorphism in the regional density of NKB fibers and the incidence of their appositions to GnRH-IR cells (101). A further interesting sex difference occurred in the overlap between KP-IR and NKB-IR inputs to GnRH neurons; immunofluorescent studies identified a higher percentage of NKB-IR afferents with KP in women (31%) compared with men (9%). The percentage of KP-IR contacts co-containing NKB was also higher in females (26%) vs. males (10%). A different immunohistochemical study (228) identified additional aspects of a sexual dimorphism of the human NKB system. The NKB-IR innervation of the Inf in this study was higher in adult females compared with males, whereas the pars tuberalis received a dense NKB-IR innervation in adult males but not in females (228). In addition, the volume occupied by NKB immunoreactivity in the Inf of adult men was significantly lower compared with adult women and with adult male-to-female transsexuals (228).

### Aging-dependent changes of the kisspeptin and neurokinin B systems in men

Aging-related decline in reproductive functions is less dramatic in men than in women because of the sustained testosterone production by the testes (229), although the negative feedback response of the reproductive axis to testosterone shows a declining trend with aging (230). Clinical symptoms of late-onset hypogonadism in elderly men include decreased morning erections, erectile dysfunction, and decreased frequency of sexual thoughts (231). Based on animal experiments, KP and NKB neurons play an important role in testosterone negative feedback to the male hypothalamus (165, 174). From this, we anticipated enhanced central KP- and NKB-signaling in the Inf of aged *vs*. young men. We have carried out quantitative immunohistochemical studies on a relatively large number (*N* = 20) of hypothalamic samples from adult men (49) and analyzed the same immunohistochemical parameters that showed sexual dimorphism in aged subjects.

This study identified robust aging-dependent enhancements in the regional densities of KP-IR perikarya and fibers, and in the incidence of contacts they established with the cell bodies and dendrites of GnRH neurons (49). The number of NKB-IR perikarya, the density of NKB-IR fibers and the incidences of axonal appositions to GnRH neurons also increased with age, but to lesser extent than in case of KP (49). In addition, in dual-immunofluorescent studies, the incidence of NKB-IR perikarya that co-contained KP increased from 36% in young to 68% in aged men, indicating that a higher proportion of NKB neurons express detectable levels of KP in aged individuals (49). Finally, we identified a mild but statistically significant hypertrophy of KP-IR and NKB-IR neurons. The magnitude of the increase was reminiscent to the hypertrophy of unidentified neurons in the Inf of aged men (232). It requires clarification if the increased incidences of KP-IR and NKB-IR contacts on GnRH neurons in aged male individuals (49) reflect increased numbers of synapses or alternatively, enhanced detectability of KP-IR and NKB-IR fibers. The first possibility gains support from the electron microscopic observation on male rat that the number of axo-somatic synapses to GnRH neurons increases about threefold by middle age and 10-fold by old age (233).

## Important Remaining Issues

Morphological studies of post mortem human hypothalami will remain valuable tools to address further important questions unanswered so far. The aims of such studies will include:

1. Identification of GnRH neuron subpopulations based on anatomical criteria.
2. 3-D reconstruction of the GnRH neuron with its dendritic arbor.
3. Identification and subcellular localization of neuropeptide receptors (KISS1R; NK3, etc.) in GnRH neurons.
4. Characterization of neuroanatomical changes in the GnRH network in association with pubertal transition.
5. Identification of anatomical changes in the GnRH network accompanying reproductive aging.
6. Anatomical and neurochemical characterization of the two human KP cell populations.
7. Further analysis of sex steroid feedback sites, including the Inf and the putative human “AVPV” (“RP3V”), and characterization of steroid target cells.
8. Clear distinction of possible organizational sex steroid effects that may contribute to the sexual dimorphism of the human KP neuronal network.
9. Localization of steroid hormone receptors in human KP neurons.
10. Identification of new hypothalamic and extrahypothalamic target cells to KP neurons.
11. Characterization of the afferent connectivity of KP neurons.
12. Identification and characterization of new players in the pulse generator.

## Conflict of Interest Statement

The authors declare that the research was conducted in the absence of any commercial or financial relationships that could be construed as a potential conflict of interest.

## Abbreviations

ARC, arcuate nucleus; CRH, Corticotropin-releasing hormone; E2, 17β-estradiol; ERα, α estrogen receptor isoform; ERβ, β estrogen receptor isoform; FSH, follicle stimulating hormone; GABA, γ-aminobutyric acid; GABA_A_/GABA_B_, Type A, B GABA receptor; Gal-R1, type-I galanin receptor; GFP, green fluorescent protein; GnIH, gonadotropin-inhibiting hormone; GnRH, type-I gonadotropin-releasing hormone; GPR147, RFRP(GnIH) receptor; Inf, infundibular nucleus; InfS, infundibular stalk; IR, immunoreactive; KA2, kainate receptor-2; KCC2, K-Cl co-transporter; KISS1R, kisspeptin receptor; KNDy, kisspeptin/neurokinin B/dynorphin; KP, kisspeptin; LH, luteinizing hormone; mRNA, messenger ribonucleic acid; NK1-3, neurokinin receptor, type 1-3; NKB, neurokinin B; NPY, neuropeptide Y; NR1, *N*-methyl-d-aspartate receptor-1; OVLT, organum vasculosum of the lamina terminalis; RFRP, RF-amide related peptide; RP3V, rostral periventricular area of the third ventricle; RT-PCR, reverse transcription-polymerase chain reaction; SP, Substance P; VGLUT1/VGLUT2, Type-1/Type-2 vesicular glutamate transporter; VIAAT, vesicular inhibitory amino acid transporter; Y5, Type 5 NPY receptor.
